# Determination of the one-electron reduced Wigner function for a molecular crystal

**DOI:** 10.1107/S2053273326009678

**Published:** 2026-10-09

**Authors:** Sizhuo Yu, Jean-Michel Gillet

**Affiliations:** a Université Paris-Saclay, CentraleSupélec, CNRS, Laboratoire SPMS, 91190, Gif-sur-Yvette, France; University of Warsaw, Poland

**Keywords:** quantum crystallography, quantum chemistry, quantum physics, Wigner function

## Abstract

The Wigner function provides a complete phase-space representation of a quantum state. Yet, direct experimental access to such a quantity for electrons in crystalline solids remains an elusive goal. Here, we report the first reconstruction of an experimental one-electron Wigner function in a molecular crystal. By jointly refining an ensemble *N*-representable model against high-resolution X-ray diffraction structure factors and directional Compton profiles, we reconstruct a 6D phase-space distribution consistent with both position- and momentum-space data. The reconstructed Wigner function exhibits pronounced negative regions that provide a direct indication of the non-classical nature of the electronic state and shows remarkable agreement with correlated post-Hartree–Fock calculations. Our results demonstrate that complementary scattering experiments can be unified within a single phase-space tomography approach, opening a route towards experimentally reconstructed electron quantum states in crystalline materials.

## Abstract 2

The first reconstruction of an experimental one-particle reduced Wigner function for electrons in molecular crystals is reported. It relies on an ensemble *N*-representable model jointly refined from the X-ray diffraction and Compton scattering data.

## 1. Introduction

Classical statistical physics was built in phase space, where a system is described by the positions 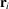 and momenta 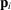 of its components (Nolte, 2010 ▸). With the advent of quantum mechanics, the incompatibility of these quantities appeared to preclude such a representation. Yet, if classical physics arises as a limit of the quantum world, a phase-space formulation must remain. E. Wigner opened this possibility (Wigner, 1932 ▸) by introducing the function 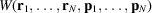 as a phase-space analogue of the *N*-particle wavefunction 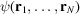. Because this 6-*N*-dimensional object is intractable for electrons even in the simplest molecular system, practical use focuses on its reduced forms: the two-electron Wigner function 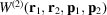 and its one-electron version 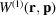, hereafter denoted 1-Wigner.

Being formally equivalent to the one-electron reduced density matrix (1-RDM), the 1-Wigner allows a complete quantum description on the one-electron level. From an experimental perspective, quantum state tomography is now routinely used to reconstruct Wigner functions of photons and atoms (Lvovsky & Raymer, 2009 ▸; Smithey *et al.*, 1993 ▸; Leibfried *et al.*, 1996 ▸), providing direct visualization of non-classical phenomena. However, achieving an analogous phase-space tomography for interacting fermions, specifically extracting the 1-Wigner for chemical bonds within a crystalline environment, has remained a formidable challenge.

Generally, the experimental investigation of electrons in crystals relies on distinct scattering techniques: high-resolution X-ray diffraction (Macchi *et al.*, 2015 ▸) [or electron diffraction (Genoni *et al.*, 2018 ▸)] yields precise position-space densities 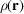, while Compton scattering provides momentum-space densities 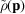 (Cooper, 1997 ▸). Because each method alone measures only a projection of the full phase-space distribution, combining them to reconstruct the underlying quantum state constitutes a challenging inverse problem. The possibility of directly extracting such a description from X-ray data was first proposed by Clinton, Massa and co-workers in a density-matrix formulation (Clinton *et al.*, 1969 ▸; Clinton *et al.*, 1973 ▸), whose iterative scheme drives the 1-RDM towards idempotency and thereby restricts the solution to a single-determinant wavefunction. Schmider, Smith and Weyrich subsequently showed that position- and momentum-space data can be combined to constrain the same object (Schmider *et al.*, 1990 ▸; Schmider *et al.*, 1992*b* ▸; Schmider *et al.*, 1993 ▸), and examined its phase-space content through the zero-momentum cut of the 1-Wigner (Schmider, 1996 ▸). The present approach departs from this lineage in replacing idempotency by the weaker ensemble *N*-representability conditions, which do not exclude fractional occupations and hence electron correlation, and in formulating the joint reconstruction as a constrained convex optimization problem. Following advances on its theoretical formulation (Foley & Mazziotti, 2012 ▸; De Bruyne & Gillet, 2020 ▸; Sizov & Staroverov, 2024 ▸), this problem has been recently revisited, demonstrating that a full reconstruction can now be envisaged for actual molecular crystals (Yu & Gillet, 2024 ▸; Yu & Gillet, 2025*a* ▸; Yu & Gillet, 2025*b* ▸; Matta, 2025 ▸).

This work reports on the first experimentally reconstructed one-electron reduced Wigner function (1-Wigner) for electrons in a molecular crystal. The reconstruction is formulated as an inverse problem, in which an ensemble *N*-representable phase-space model is constrained by complementary crystallographic data from high-resolution X-ray diffraction and Compton scattering, each probing a marginal of the 1-Wigner function. This procedure constitutes a phase-space quantum crystallographic tomography (PS-QCT) of electrons in crystals, which we demonstrate on the molecular crystalline system of urea. Section 2 introduces the Wigner function and its relation to position- and momentum-space observables. Section 3 gives the reconstruction model and ensemble *N*-representability conditions. Section 4 discusses the treatment of experimental data and shows the principal results on the urea crystal. Section 5 concludes by discussing the implications and limitations of the method.

## 2. The 1-Wigner and its projections to measurable quantities

For a pure-state *N*-electron wavefunction ψ, the 1-Wigner function can be expressed as 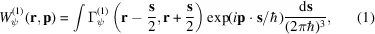
where 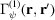 is the one-electron reduced density matrix in position representation (1-RDM), 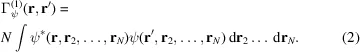
The following formulations extend naturally to mixed states and spins by linearity. Therefore, we will omit explicit reference to the state ψ hereafter. From its definition, the 1-Wigner 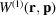 is directly related to the position- and momentum-space electron densities (O’Connell, 1983 ▸):
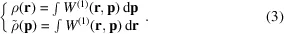
As a consequence, the most obvious experimental information for position space, which can be obtained on the 1-Wigner, is expected to arise from X-ray (or electron) diffraction structure factors. For this work, only X-ray diffraction is considered. For such an elastic coherent scattering process, the signal at the Bragg condition is proportional to the modulus squared of the structure factors, *i.e.*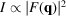, where 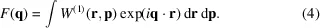
In other words, X-ray diffraction measures the position-space marginal density’s phaseless Fourier coefficients. Additionally, since the experimentally observed structure factors necessarily include thermal motion contributions, appropriate modelling is required to recover the phases and correct for the thermal effects (Appendix *B*[App appb]).

By contrast, deep inelastic X-ray scattering directly probes projections of 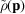 through directional Compton profiles 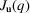, given in the impulse approximation (Platzman & Tzoar, 1965 ▸) by 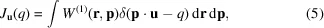
where the unit vector 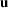 points in the scattering vector direction and will be indicated by the triplet *hkl*.

Over the past decades, high-resolution diffraction data have enabled accurate reconstructions of 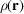 (Gatti & Macchi, 2012 ▸), while extensive sets of Compton profiles have yielded 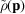 (Cooper, 1997 ▸). Since both 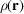 and 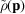 are 3D projections of the 6D 1-Wigner [equation (3)], the reconstruction of the latter is intrinsically a tomographic problem. In contrast to quantum-state tomography applied to photon beams (Lvovsky & Raymer, 2009 ▸), molecular motions (Zhang *et al.*, 2021 ▸) or ballistic electrons (Jullien *et al.*, 2014 ▸), electrons in crystals can only be probed in limited configurations allowed by the available scattering experiments. The reconstruction of the 1-Wigner thus necessitates the use of a parametric model.

## 3. The ensemble *N*-representable model for reconstruction

For a one-electron phase-space representation to be physically valid, it must be able to represent either an *N*-electron wavefunction or a statistical mixture of such wavefunctions (Coleman, 1963 ▸; Parr & Yang, 1989 ▸). In a series of seminal papers, Clinton and Massa (Clinton *et al.*, 1969 ▸; Clinton *et al.*, 1973 ▸), but also Aleksandrov *et al.* (1989 ▸), chose to enforce pure-state *N*-representability by constraining the very strict idempotency condition of their 1-RDM. However, an idempotent 1-RDM can only come from a single-determinant wavefunction. It is thus associated with a mean-field model. In the present work, as suggested by the series of works of Schmider, Weyrich and Smith (Schmider *et al.*, 1990 ▸; Schmider *et al.*, 1992*a* ▸; Schmider *et al.*, 1992*b* ▸; Schmider *et al.*, 1993 ▸), no such restriction is required, as the more general ensemble *N*-representability conditions can be applied via the usage of constrained convex optimization. Our model expands the 1-Wigner on an orthogonal basis of atomic functions 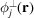, so that 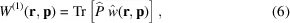
where 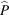 is the unknown matrix and the elements of 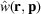 are fully determined by the basis functions: 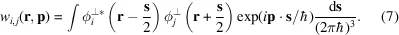
Without loss of generality, we shall omit the specific usage of 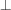 in the following, and assume that all basis functions are orthonormal. Non-orthogonal basis functions can be orthogonalized using Löwdin’s procedure (Löwdin, 1950 ▸). Ensemble *N*-representability is enforced on the matrix 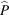 as 
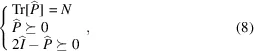
assuming a paired-electron system. Equation (8) ensures that the eigenvalues of 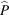, *i.e.* the occupation numbers of natural orbitals, lie between 0 and 2 (Coleman, 1963 ▸). The single-determinant limit corresponds to eigenvalues of the *P*-matrix being either zero or 2. For a spin-polarized electron gas, separate *P*-matrices could be introduced for each spin state. In this work, the orthogonal atomic basis is derived from a standard Pople 6-31G set augmented with a p-type polarization function on hydrogen atoms (Levine, 2014 ▸), with frozen cores and full symmetry treatment. Model validation and reconstruction reliability, particularly from the 1-RDM’s perspective, have been discussed elsewhere (Yu & Gillet, 2024 ▸; Yu & Gillet, 2025*a* ▸; Yu & Gillet, 2025*b* ▸; Matta, 2025 ▸).

The choice of basis set is motivated by the stability of the reconstruction, a primary consideration for this first-of-its-kind experimental determination of the 1-Wigner function; the analysis of basis-set effects is deferred to a further study.

Once an orthogonal atomic basis is defined, the 1-Wigner model is fully specified by equation (6) under the constraints of equation (8). The symmetric *P*-matrix elements, which constitute the unknowns of the reconstruction, are determined by minimizing a 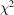 objective function based on the available experimental data (see Appendices *A*[App appa] and *D*[App appd]). Both this objective function and the *N*-representability conditions are convex, so that the refinement is a constrained convex optimization problem: the solver converges to the global optimum and returns the same solution irrespective of the starting point. Two further constraints are imposed in practice, namely the symmetry adaptation of the orbitals and a frozen-core condition, which are detailed in Appendix *D*[App appd]. The residual errors are therefore not of algorithmic origin, but originate from the experiment itself, from the data treatment, and from the truncation of the model to a finite basis; their combined effect is difficult to quantify rigorously, and we assess it here through the global indicators reported in the supporting information.

## 4. Results

To validate the PS-QCT method, we selected urea [CO(NH_2_)_2_] as the test system, whose molecular structure is shown in the inset of Fig. 1 ▸. Urea serves as the archetype of quantum crystallography. As a molecular crystal, it permits well defined molecule-based modelling within a periodic lattice, while its chemically rich bonding provides a stringent and physically meaningful test of electron-density and phase-space reconstruction methods. We utilize the directional Compton profiles (DCP) and high-resolution synchrotron X-ray diffraction datasets reported by Reed *et al.* (1978 ▸), Shukla *et al.* (2001 ▸) and Birkedal *et al.* (2004 ▸). These datasets remain the benchmark for this system due to their exceptional momentum resolution (0.1 a.u.) and high-order diffraction scattering vectors (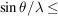 1.44 Å^−1^), while providing the necessary statistics to resolve subtle electron correlation effects in phase space (Hupf *et al.*, 2023 ▸; Erba *et al.*, 2010 ▸). For the X-ray diffraction dataset, a Hansen–Coppens model is used by the *MoPro* software to obtain the phased and thermally deconvoluted structure factors (SF) (Hansen & Coppens, 1978 ▸; Jelsch *et al.*, 2005 ▸). For the higher-resolution Compton data, only anisotropies were published (Shukla *et al.*, 2001 ▸), and the original full profiles have been lost (Shukla, 2025 ▸). Thus, three full DCPs were reconstructed by combining the deconvoluted DCP along the [100] direction reported by Reed *et al.* (1978 ▸) and two anisotropies published by Shukla *et al.* (2001 ▸). The details of these treatments are further explained in Appendices *B*[App appb] and *C*[App appc].

The reconstructed 1-Wigner of urea is shown in Fig. 1 ▸. Since 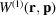 resides in a 6D phase space, we visualize it via a 2D cut 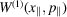. Here, the position coordinate follows the O–C–N–H nuclei path (inset of Fig. 1 ▸) to highlight the specific electronic structure of the bonding regions. The momentum coordinate 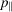 represents the projection of 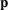 onto the unit vector tangent to the path at each position 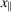. Two important features can be identified in the reconstructed 1-Wigner: the coexistence of positive and negative regions, which distinguishes the 1-Wigner as a quasi-probability function from a classical probability distribution in phase space, and the positive stripe-like patterns that appear along the momentum direction, centred on each (non-hydrogen) atomic site. These stripes originate from the core electron contributions, which are localized in the vicinity of the nuclei’s positions and therefore expand more broadly in momentum space. Conversely, but still in line with Heisenberg’s inequalities, low-momentum regions with successive negative and positive values spread over a broad range of position space and correspond to the σ valence electrons involved in the chemical bond. The path indeed lies in the molecular plane, which is the nodal plane of the π orbitals, so that the present cut is essentially free of π contributions. Many of these features can be connected to Schmider’s analysis of the 1-Wigner’s zero-momentum cut (Schmider, 1996 ▸). Schmider’s paper demonstrates that chemical bonding signatures are encoded in the phase-space distribution, providing a framework for interpreting the low-momentum limit of our reconstruction as the quantum expectation value of the local parity operator.

The subtle effects induced by the formation of chemical bonds are better seen on the deformation 1-Wigner 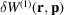, defined as 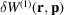 = 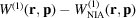, where 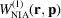 is the 1-Wigner computed from the contribution of its non-interacting atoms. Fig. 2 ▸ compares the reconstructed deformation 1-Wigner with an *abinitio* calculation performed at the post-Hartree–Fock (CCSD) level with an external polarization field to account for the crystal environment. It uses the same basis set as the reconstruction model to avoid introducing basis-set effects into the comparison. Here, again, the most obvious features of the formation of a chemical bond can be seen at low momenta (between 0 and 2 a.u.) and are characterized by a large position delocalization, typical of what is expected from valence electrons. The reconstructed and theoretical 1-Wigner functions show excellent agreement, confirming that the tomography captures the phase-space manifestation of chemical bond formation.

The deformation 1-Wigner clearly illustrates the need to include momentum-space Compton measurements as an additional source of information. Reconstructions based solely on X-ray diffraction structure factors exhibit shapes of positive and negative regions that differ significantly from theoretical predictions, highlighting the critical role of a joint position–momentum refinement.

From a pure position-space perspective, the reconstruction quality is better seen by comparing the deformation density [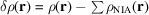], where the promolecular density is computed using Clementi–Roetti functions (Clementi & Roetti, 1974 ▸). The multipolar deformation density yields a description of chemical bonding that is essentially consistent with the *abinitio* calculation results [Fig. 3 ▸ (A1) versus (A2)] (Jelsch *et al.*, 2005 ▸). The most prominent discrepancy is observed at the O–C bond, where the multipolar deformation density lacks the negative contribution found both in the *ab initio* calculation and in the two reconstructions. Remarkably, as shown in Fig. 3 ▸ (B1) and (B2), deformation densities derived from the reconstructed 1-Wigner (refined against the *MoPro* static SF) align more closely with theory than the *MoPro* density itself. This likely stems from the model utilizing the same basis functions as the *abinitio* calculations, which implicitly constrains the refinement. Finally, the inclusion of DCP data in the refinement exerts a noticeable but minor influence on the position-space density.

We now turn to the momentum space, as we plot the Compton profile (CP) anisotropies in Fig. 4 ▸. The CP anisotropies represent the differences between CPs in different crystallographic directions. The fine structures in the anisotropies are known to be highly sensitive to electron correlation and crystal field effects (Shukla *et al.*, 2001 ▸; Erba *et al.*, 2010 ▸). For the urea crystal, the strongest anisotropy is found between the [001] and [110] directions, although peaking at only 4% of the total DCP maximum. Notably, despite relying on a molecular-based model, the CP anisotropies derived from the reconstructed Wigner function successfully reproduce all the subtleties of these weak features seen in the experimental Compton spectra. As expected, we observe that this level of agreement could not be reached when only X-ray diffraction data are included in the reconstruction.

## 5. Discussion and conclusion

The possibility of experimentally reconstructing the 1-Wigner of electrons carries several theoretical implications. More general than the momentum- and position-space densities as separate entities, the 1-Wigner enables the direct determination of the eigenvalues of the one-electron density matrix, namely the natural-orbital occupation numbers (NON). Whereas the idempotency condition underlying a single-determinant description restricts the NON to 0 or 2, the ensemble *N*-representable model admits any value in between: fractional occupations, which quantify electron correlation beyond a mean-field description, thus become experimentally accessible. We nonetheless refrain from interpreting the NON of the present reconstruction in these terms, urea being too weakly correlated for such an analysis to be conclusive given the presence of experimental noise and modelling error (Yu & Gillet, 2025*b* ▸). Moreover, nontrivial structure in the occupation spectrum, such as pinning and quasipinning of generalized Pauli constraints, would become, at least conceptually, accessible to direct experimental scrutiny (Schilling, 2015 ▸). Reduced density-matrix functional theory (RDMFT), whose fundamental variable is the ground-state 1-RDM, could similarly gain from experimentally validated 1-RDMs and occupation-number spectra, aiding both functional development and assessments in strongly correlated fermionic systems (Di Sabatino *et al.*, 2015 ▸).

Although urea is used here as a demonstration, owing to its experimental data availability and moderate size, the underlying phase-space tomography framework is not restricted to weakly correlated systems. The reconstruction targets the 1-RDM, whose dimensionality and associated optimization complexity depend on the chosen one-particle basis set rather than on the degree of electronic correlation. In this sense, strong correlation does not increase the formal complexity of the reconstruction.

The extension to other crystals is, however, presently limited by the availability of data rather than by the method itself, since both high-resolution X-ray diffraction and directional Compton profiles are required for the same compound. Among molecular crystals, only urea and ice Ih currently meet this condition. Going beyond molecular crystals raises a further difficulty: the periodicity would have to be carried by the model itself, through Bloch functions, at the cost of a considerable increase in the number of free parameters. A correspondingly more constrained model would then be needed to keep the reconstruction stable, and this is the direction we are currently exploring.

At first glance, reconstructing the 1-Wigner function from partial knowledge of its two marginals (the position-space density from structure factors, the momentum-space density from directional Compton profiles) appears underdetermined: a 6D object inferred from incomplete information on two of its 3D projections. This holds for a model-free reconstruction, carried out point by point in phase space, which is not what is attempted here. Because the 1-Wigner is parametrized by the population matrix of a finite basis set of size *K*, the unknowns reduce to the 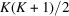 independent elements of a real symmetric matrix, fewer still once the block-diagonal symmetry and frozen-core constraints are imposed. These parameters are determined from a larger number *M* of measurements by minimizing a convex objective function under the ensemble *N*-representability constraints.

This provides, in principle, a rationale for reconstructing the population matrix. However, this does not yet amount to an answer to the uniqueness, which would require establishing when the map from the population matrix to the observables is injective. This question has been studied in its own right, but so far under the assumption of exactly known densities (Harriman, 1986 ▸; Harriman, 1993 ▸; Sizov & Staroverov, 2024 ▸); its counterpart for incomplete and noisy scattering data is the subject of a separate publication (Yu & Gillet, 2026 ▸). The present work shows that such convex refinement, when applied to experimental data, yields a 1-Wigner function in close agreement with theoretical predictions and remains stable over a wide range of reconstruction parameters (see the supporting information).

This work demonstrates that our 1-Wigner model provides a unified framework for jointly refining electronic structure using information from complementary scattering experiments. In our approach, both X-ray diffraction structure factors and directional Compton profiles are integrated consistently into the reconstruction of an ensemble *N*-representable 1-Wigner. The mutual compatibility of both representations of electron density is thereby guaranteed by construction. Beyond its marginal distributions, the reconstructed experimental 1-Wigner function [defined as the expectation value of a parity operator in phase space (Royer, 1977 ▸)] reveals pronounced negative regions that provide a direct indication of the non-classical nature of the electronic state (Kenfack & Życzkowski, 2004 ▸; Hudson, 1974 ▸). Their shapes and amplitudes align closely with those obtained from a post-Hartree–Fock calculation. Our results show that this tomography achieves at least deformation-level accuracy and captures the essential features associated with chemical bond formation.

Elastic and inelastic X-ray scattering studies have long pursued distinct objectives, focusing on charge-density and bonding in the former case and on collective excitations in the latter. Yet both probe the same electrons, and their combination enables a far richer quantum description than either alone. By demonstrating the feasibility of phase-space quantum crystallographic tomography (PS-QCT) as a unified reconstruction approach, we aim to foster coordinated experimental efforts in which a single phase-space reconstruction not only gives access to a complete characterization of electronic behaviour in crystals, but also certifies the mutual consistency of its position- and momentum-space representations.

## Supplementary Material

Supporting information. DOI: 10.1107/S2053273326009678/pl5056sup1.pdf

## Figures and Tables

**Figure 1:**
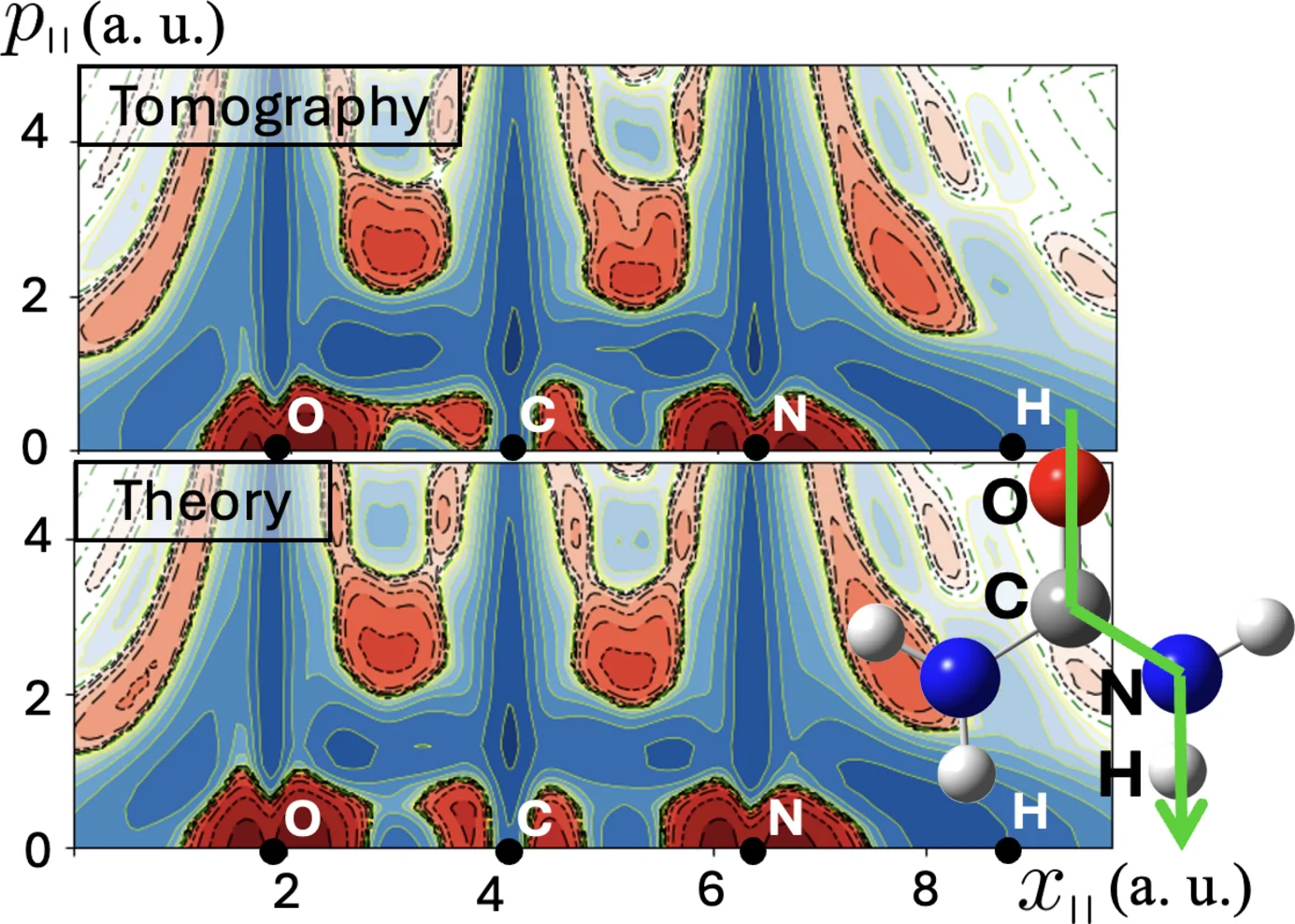
Experimentally reconstructed 1-Wigner 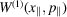 from PS-QCT (top), compared with the corresponding theoretical results (bottom). This is a 2D cut of the 6D phase space, and not a map of position space: the horizontal axis is a position coordinate and the vertical axis a momentum coordinate. The position coordinate 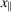 follows the O–C–N–H path in the urea molecule (inset), the nuclei being marked by black dots. The momentum direction 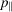 is chosen to be tangent to the path for each curvilinear point 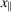. Contours are every 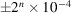 a.u., for positive (blue, solid line) and negative (red, dashed line) values, and green dash–dotted contours are drawn for zeros. All panels share the same contour levels.

**Figure 2:**
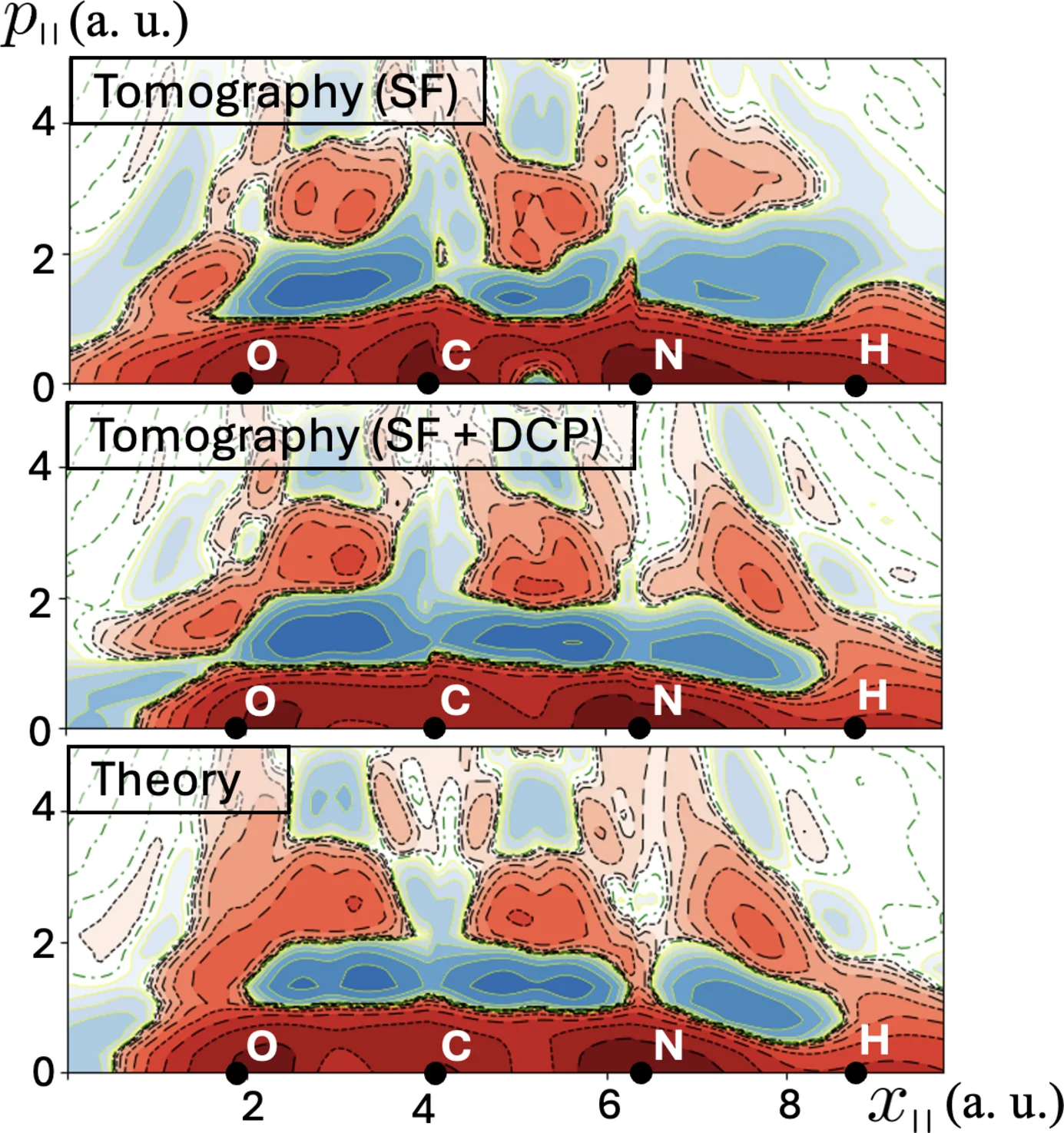
Comparison between PS-QCT and theoretical deformation 1-Wigner. Top: 1-Wigner from PS-QCT using only X-ray diffraction data. Middle: 1-Wigner from PS-QCT using both diffraction and Compton scattering data. Bottom: theoretical 1-Wigner at 6-31G[H(p)]/CCSD level with polarization field. The deformation 1-Wigner is defined as the total 1-Wigner minus the sum of independent atomic 1-Wigners (see text for definition). The 2D cut is the same as Fig. 1 ▸: the horizontal axis is the position along the O–C–N–H path and the vertical axis the momentum component tangent to that path. Contours are every 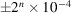 a.u., for positive (blue, solid line) and negative (red, dashed line) values, and green dash–dotted contours are drawn for zeros. All panels share the same contour levels.

**Figure 3:**
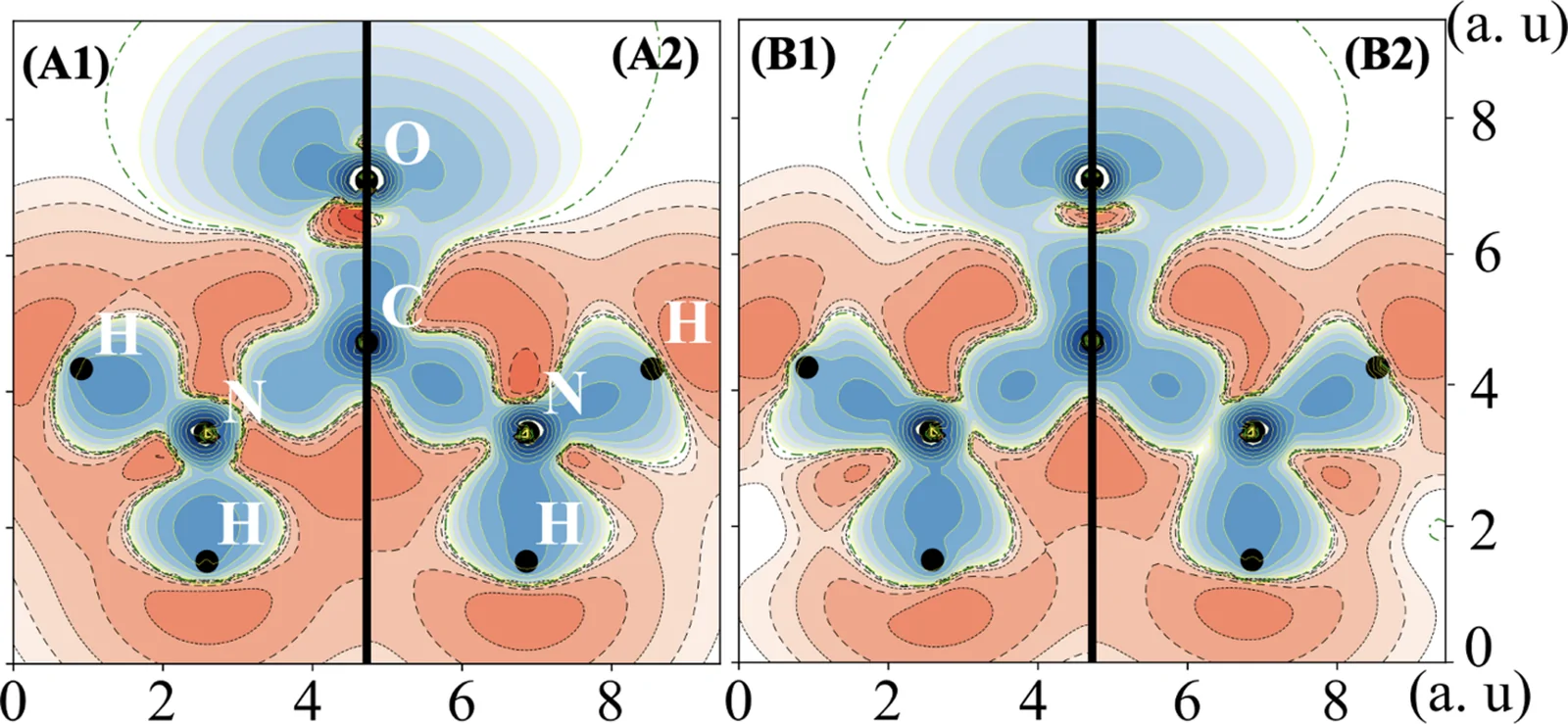
Position-space deformation densities on the molecular plane: each of the two panels is split by a vertical line into two half-maps, the symmetry of the urea molecule allowing two densities to be compared side by side. (A1): the deformation density from theoretical calculation at 6-31G[H(p)]/CCSD level with polarization field. (A2): the deformation density obtained from multipolar refinement used in data treatment with *MoPro* software. (B1): reconstruction with SF data alone. (B2): reconstruction from the joint use of SF and DCP data. Contours are every 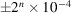 a.u., for positive (blue, solid line) and negative (red, dashed line) values, and green dash–dotted contours are drawn for zeros. All panels share the same contour levels.

**Figure 4:**
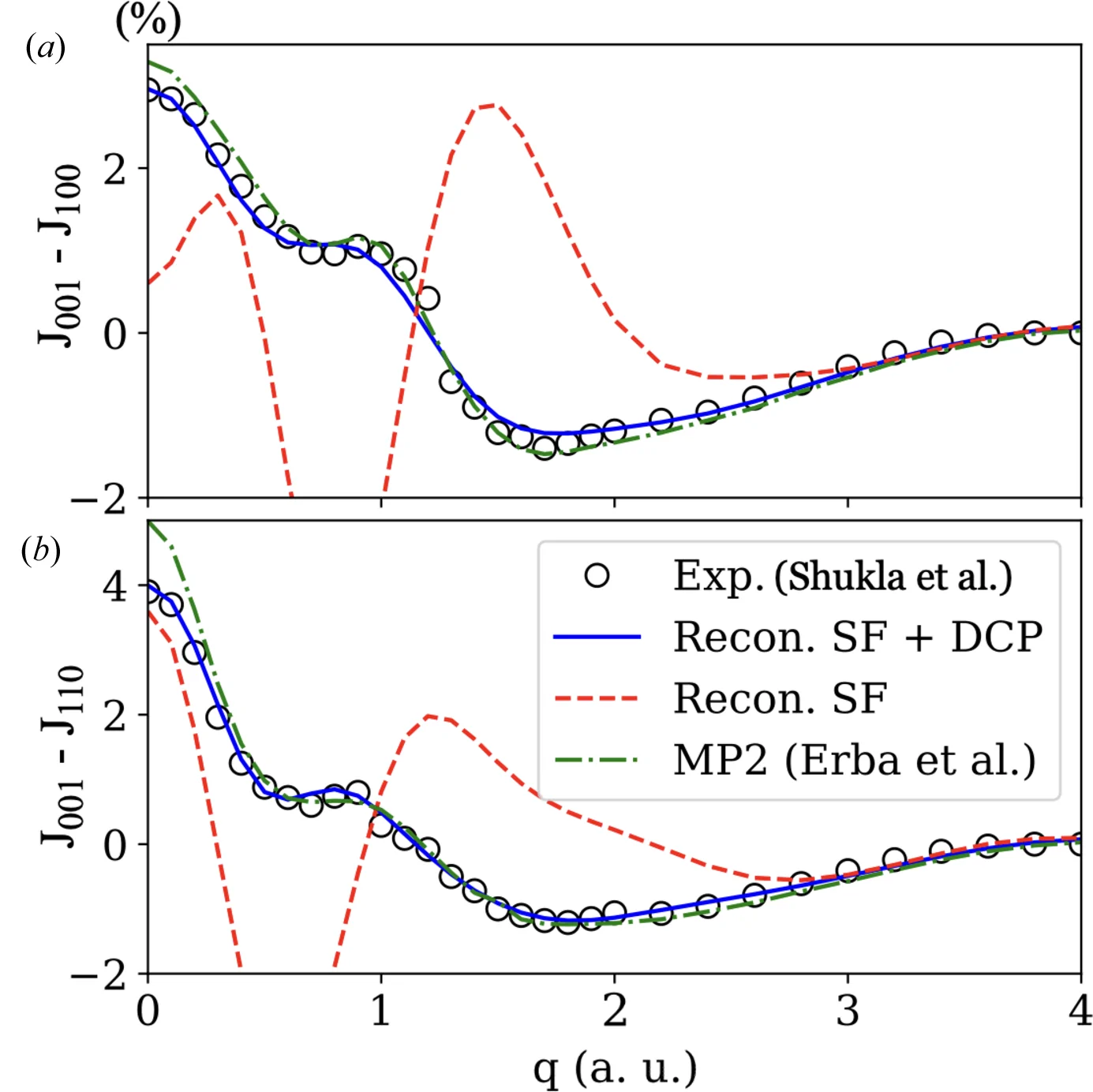
Directional Compton anisotropies. (*a*) The anisotropy between 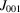 and 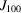. (*b*) The anisotropy between 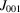 and 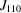. Different data, including synchrotron experiment (dots), 1-Wigner reconstruction using SF and DCP (solid blue line), 1-Wigner reconstruction using SF alone (dashed red line) and theoretical values (dash–dotted green line) are given as a percentage of 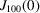.

## Data Availability

The raw X-ray diffraction structure factors (XRSF) were obtained from the published work of Birkedal *et al.* (2004 ▸). The XRSF were then treated by the *MoPro* software to recover the phase factors and to correct for the thermal vibration effect (Jelsch *et al.*, 2005 ▸). The directional Compton profiles (DCPs) were combined from two separate published experiments by Shukla *et al.* (2001 ▸) and Reed *et al.* (1978 ▸). More specifically, since the original high-resolution full DCP data of Shukla *et al.* have been lost, we have combined their high-resolution DCP anisotropies with the [100] direction measured by Reed *et al.* to recover the full DCP. The refinement program, developed by the authors, relies on the *MOSEK* optimization software (MOSEK ApS, 2026 ▸) and the *CVXPY* interface (Diamond & Boyd, 2016 ▸; Agrawal *et al.*, 2018 ▸). The theoretical *ab**initio* molecular quantum chemistry calculations were performed with the *PySCF* package (Sun *et al.*, 2018 ▸; Sun *et al.*, 2020 ▸).

## Appendix A Calculation of observables’ integrals

With definition (1), all the quantum information included in the wavefunction(s) can be accounted for when computing one-electron observable expectation values by a mere integral in phase space,

provided that the phase-space representation of the observable  (as we save the hatted notation  for the matrix representation) is defined as the Weyl transform:

It is equivalent and perhaps more straightforward to compute these observables directly using the 1-RDM function expressed in some basis set , *i.e.*

where  is the population matrix.  in the discrete basis-set representation is defined as

### A1. Structure factors

The structure factors are the Fourier coefficients of the position-space density:

Hence the discrete matrix representation:

### A2. Directional Compton profiles

Directional Compton profiles are the projections of the momentum-space density:

Here, by convention,  is the normalized vector defining the direction of the reciprocal vector and *q* its norm. One way of computing its matrix elements from the basis functions in their position representation is to first compute the auto-correlation function . The associated matrix elements are thus

whose Fourier transform gives the momentum-space density. Then, one finds

We note that the normalization terms in (16) and (19) have been ignored. By definition, they are normalized to the number of electrons so that  and .

## Appendix B Multipolar refinement

In the current work, the 2024 version of the *MoPro* software (Jelsch *et al.*, 2005 ▸) was used to perform the position-space refinement and correction for the SF. The *MoPro* software is a modern implementation of the Hansen–Coppens multipolar model with thermal modelling within and beyond the harmonic approximations. A  refinement is carried out using multipoles up to octupoles for non-hydrogen atoms and up to dipoles for hydrogen atoms.

## Appendix C Data treatment of the directional Compton profile data

Unlike SF, DCPs are largely immune to the thermal motion problem. In the impulse approximation, a DCP represents the projection of the momentum-space density, where thermal effects at typical crystallographic temperatures can be safely neglected.

For urea, two sets of DCP data were available: a lower-resolution dataset ( a.u.) collected using a laboratory-based γ-ray source (Reed *et al.*, 1978 ▸), and a higher-resolution dataset ( a.u.) obtained by Shukla *et al.* (2001 ▸) using a synchrotron source. However, only Compton profile anisotropies have been published from the latter study – the differences between the DCPs.

To fully utilize the high-resolution synchrotron data, we combined the two datasets. The low-resolution DCP along the [100] direction, , was selected as the base profile, as it is reported to be closest to the spherically averaged Compton profile. The two anisotropies from the high-resolution study,

were then added to this base to reconstruct the high-resolution directional profiles:

Together with the original , these three reconstructed DCPs form the combined dataset used to reconstruct the 1-Wigner function reported in this paper. The validity of this recombination procedure is assessed in the supporting information, by comparing the reconstructions obtained from the low-resolution total profiles alone and from the recombined high-resolution profiles.

## Appendix D Refinement with constrained convex optimization

After obtaining the experimental phased, static SF from the *MoPro* procedure, the 1-Wigner model is refined against both SF and DCP data. More specifically, a  function is minimized, *i.e.*

where  is the number of data points and  the effective degrees of freedom of the model. Since the term  does not affect the global minimum, it will be omitted from the discussion. The  and  are, respectively, the observable *A*’s experimental measurement *i*th value and estimated associated quadratic error, while  is the corresponding prediction from the 1-Wigner model. The parameters to be determined are the elements of the population matrix. With (13) and (22), its optimal value is given by

In addition to the minimization of the  function, the model needs to satisfy the spin-less *N*-representability conditions:

The minimized function in (23) is a convex function, and the constraints (24) are also convex. Therefore, the solution can be obtained with a constrained convex optimization solver. In the current work, we have used the *MOSEK* solver through the *CVXPY* interface (MOSEK ApS, 2026 ▸; Diamond & Boyd, 2016 ▸).

### d1. Symmetry constraints on the refinement

The wavefunction must be an eigenfunction of the symmetry operators belonging to the molecule’s symmetry group. Therefore, we can construct an auxiliary basis where all basis functions obey the molecular symmetry. It is sometimes referred to as symmetry-adapted molecular orbitals. As a consequence, the population matrix expressed in this symmetry-adapted basis has to be a block-diagonal matrix, since any product term of two basis functions belonging to different symmetry classes will break the symmetry of the model. Such constraints can be expressed as

where  is the transformation matrix to the symmetry-adapted basis.  is the set of indices of the symmetry-adapted basis functions belonging to the *a*th symmetry species.

### d2. Frozen-core constraints on the refinement

The core electrons are more stable and are usually very well described within the mean-field (Hartree–Fock) approximation. Therefore, one can effectively freeze the core electrons during the refinement by fixing the parameters associated with their molecular orbitals (MOs) from a Hartree–Fock (HF) calculation. The idea is similar to the use of an active space in quantum chemistry. Therefore, one can modify the *N*-representability constraints in (24) as

where  is the population matrix constructed from HF MOs for the core electrons. Since the HF  can take only 0 and 2 as its eigenvalues, the inequalities in (26) and the orthogonality of the basis functions together guarantee that  will be orthogonal to .
