# Supplementary material for: Determination of the one-electron reduced Wigner function for a molecular crystal
Source: Acta Crystallogr A Found Adv. 2026 Oct 9;82(Pt 6):419–27. doi: 10.1107/S2053273326009678 (PMC13720706; doi:10.1107/S2053273326009678)
Supplement: Supplementary file 1 [file a-82-00419-sup1.pdf]

Acta Cryst A
FOUNDATIONS
ADVANCES

Volume 82 (2026)

Supporting information for article:

Determination of the one-electron reduced Wigner function for a molecular crystal

Sizhuo Yu and Jean-Michel Gillet

Supplementary Information for

Determination of the one-electron reduced Wigner function for a molecular crystal

Sizhuo Yu and Jean-Michel Gillet

Université Paris-Saclay, CentraleSupélec, CNRS, Laboratoire SPMS, 91190 Gif-sur-Yvette, France

sizhuo.yu@centralesupelec.fr   jean-michel.gillet@centralesupelec.fr

Contents

S1 Energy and virial-ratio analysis of different reconstruction settings **1**

S1.1 The weighting of DCP and resolution cutoff for SF 1

S1.2 Validation of the reconstructed directional Compton profiles 3

S2 Momentum components perpendicular to the molecular plane **3**

S1 Energy and virial-ratio analysis of different reconstruction settings

In this section, we present an energy analysis of the reconstructed 1-Wigner function under different settings. While the 1-Wigner is a one-electron reduced object and therefore does not allow for a direct computation of the energy, we can construct an ansatz for the 2-RDM based on this one-body reduced description and then compute the approximate energies. This has been explained in detail in [3].

In this framework, the two-electron interaction energy is approximated, while the one-electron kinetic energy and the electron-nucleus energy are exact. Therefore, the virial ratio $-2T/V$, which equals unity for an exact non-relativistic wavefunction at the equilibrium geometry, can also be approximated and serves as a benchmark for the reconstruction quality. Both quantities are global indicators: unlike the two-dimensional cuts shown in the main text, they involve the whole reconstructed phase-space distribution.

The primary objective is to show that:

- the reconstructed 1-Wigner is stable under a wide range of reconstruction parameters;
- there is a clear improvement brought by combining the high-resolution DCP anisotropies with the low-resolution total DCP.

S1.1 The weighting of DCP and resolution cutoff for SF

In practice, the number of DCP data is not balanced with the number of SF data, therefore they are weighted in the fitting function with an empirical parameter λ, i.e.

$$\chi^2 = \chi^2_{\mathrm{SF}} + \lambda\, \chi^2_{\mathrm{DCP}}, \tag{S1}$$

where χ^2_{SF} and χ^2_{DCP} are the contributions to Eq. (22) of the main text restricted to the structure factors and to the directional Compton profiles respectively. A value $\lambda = 0$ amounts to a

refinement against the diffraction data alone. A second parameter is the resolution cutoff applied to the structure factors, i.e. the highest $\sin\theta/\lambda_X$ retained in the refinement.

Figure S1 reports the virial ratio of the reconstructed 1-Wigner as a function of λ, the solid lines corresponding to the full SF dataset and the shaded bands covering the range of SF resolution cutoffs tested.

The behaviour of the virial ratio can be understood as follows:

1. in the low-λ regime, the SF dominate the fitting; since the experimental SF carry larger errors at high reflection angle (thermal motion, signal-to-noise ratio), the full SF dataset yields the worst reconstructed 1-Wigner, and accordingly the solid lines lie at the lower edge of the bands;

2. in the mid-λ regime, the DCP balance the SF and the best overall reconstruction is achieved, while the high-angle SF data still act as a source of error;

3. in the high-λ regime, the DCP start to dominate the fitting and their systematic errors deteriorate the reconstruction; the high-angle SF data then act as a regularisation of the DCP fitting, and the solid lines move to the upper edge of the bands.

The full SF dataset thus passes from being the least to the most favourable choice as λ increases, which is the signature of the change of role of the high-angle reflections.

Taking a deviation of the virial ratio from unity of 1%, i.e. a value of 0.99, as a criterion for a good reconstruction, a wide range of λ and of resolution cutoffs is found to lie above this threshold. This is the sense in which we consider the reconstruction to be stable with respect to the reconstruction parameters.

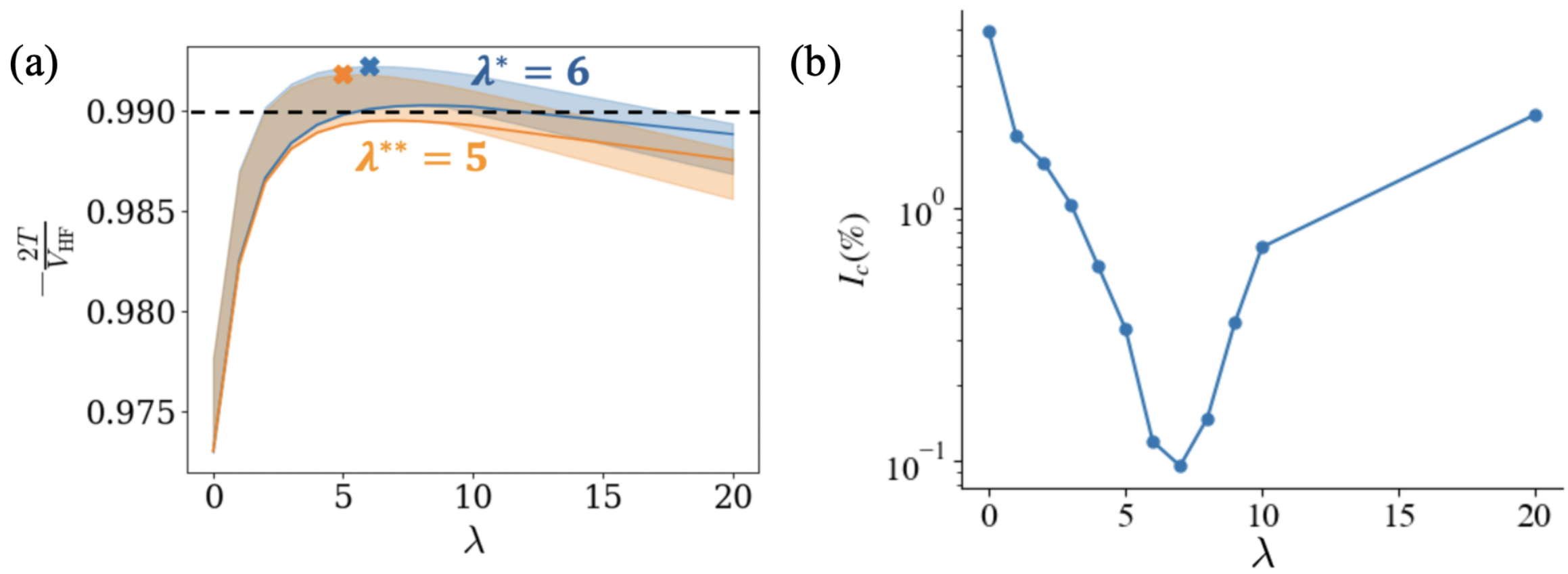

Figure S1: Dependence of the reconstructed 1-Wigner function on the DCP weighting parameter λ of Eq. (S1). (a) Virial ratio $-2T/V_{\text{HF}}$ for refinements against the recombined high-resolution DCP (blue) and the low-resolution total profiles (orange). Solid lines correspond to the full SF dataset, i.e. the highest resolution cutoff, and the shaded bands to the spread obtained over the range of cutoffs tested. Crosses mark the best reconstruction obtained for each dataset, at $\lambda^* = 6$ for the high-resolution and $\lambda^{**} = 5$ for the low-resolution data. The dashed line marks the 1% deviation from unity used as a criterion for a good reconstruction. (b) Idempotency deviation $I_c = \frac{1}{2N}\,\text{Tr}\left[2\mathbf{P}^\perp - (\mathbf{P}^\perp)^2\right]$ for the maximum SF resolution

Interestingly, the deviation from idempotency, $I_c = \frac{1}{2N}\,\text{Tr}\left[2\mathbf{P}^\perp - (\mathbf{P}^\perp)^2\right]$, also exhibits a minimum in approximately the same range of λ as that yielding the optimal virial ratio. Although electron-correlation effects are not investigated explicitly in the present work, urea is expected to be only weakly correlated. The small deviation from idempotency may therefore

be interpreted as evidence that the experimental data effectively constrain the reconstructed 1-RDM toward a nearly idempotent solution, despite the convex optimization allowing, in principle, fractional natural occupation numbers between 0 and 2.

S1.2 Validation of the reconstructed directional Compton profiles

Appendix C of the main text explains how we combine the only available high-resolution Compton profile anisotropies [2] with the low-resolution total profiles [1] to reconstruct the high-resolution DCP.

To provide evidence that the recombined DCP do take advantage from the very subtle details of the anisotropies, we can compare the virial ratio of the 1-Wigner function, reconstructed respectively against the low-resolution total profiles and the recombined high-resolution profiles. The two cases are the orange and blue curves of Fig. S1.

At their respective optima, the reconstruction using the recombined high-resolution DCP reaches a virial ratio closer to unity than the one using the low-resolution total profiles alone. Moreover, as λ increases and the DCP come to dominate the fitting, the virial ratios obtained from the high-resolution data are systematically better than those from the low-resolution one. Arguably, the difference is small, as expected from data whose distinguishing features are the weak anisotropies themselves, but it is systematic: it holds over the whole range of λ for which the Compton data effectively constrain the refinement. We take this as an indication that the recombination procedure does transfer the information carried by the high-resolution anisotropies into the reconstruction, rather than merely adding noise.

S2 Momentum components perpendicular to the molecular plane

In the main text, only a 2D partial cut of the 6D 1-Wigner function is visualised, in which the momentum coordinate is taken parallel to the position path. Here we report a different 2D cut, $W^{(1)}(x_{\parallel}, p_{\perp})$, in which the position coordinate follows the same O–C–N–H path while the momentum coordinate is taken perpendicular to the molecular plane. It reports a similar agreement between experimental reconstruction and theory, both for the total and for the deformation 1-Wigner (Fig. S2).

Together with the virial ratio, a global indicator, it reflects that although we can only partially visualise the 6D reconstructed function, the overall agreement is expected to hold elsewhere.

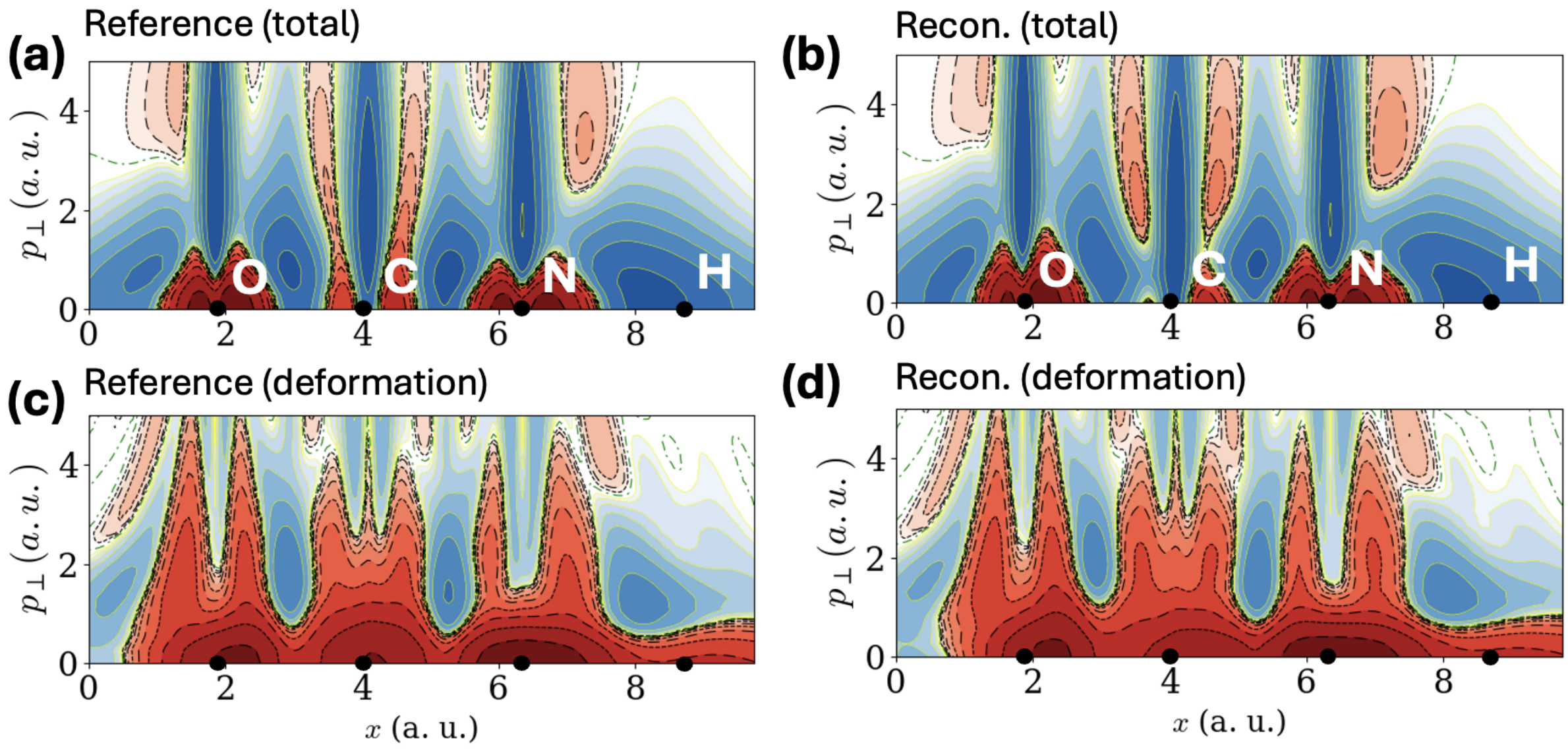

Figure S2: 1-Wigner cut $W^{(1)}(x_\parallel, p_\perp)$ with the momentum component perpendicular to the molecular plane. (a), (c): theoretical reference; (b), (d): reconstruction from the joint use of SF and DCP data. Top row: total 1-Wigner; bottom row: deformation 1-Wigner. The horizontal axis is the position along the O–C–N–H path, with the nuclei marked by black dots; the vertical axis is the momentum component perpendicular to the molecular plane. Same contour convention as Figs. 1 and 2 of the main text.

References

[1] W. A. Reed, L. C. Snyder, H. J. Guggenheim, T. A. Weber and Z. R. Wasserman, *The Compton profile of urea*, J. Chem. Phys. **69**, 288–296 (1978).

[2] A. Shukla, E. D. Isaacs, D. R. Hamann and P. M. Platzman, *Hydrogen bonding in urea*, Phys. Rev. B **64**, 052101 (2001).

[3] S. Yu and J.-M. Gillet, *N-representable one-electron reduced density matrix reconstruction with frozen core electrons*, Acta Cryst. A **80**, 249–257 (2024).
